# TGF-β1-supplemented decellularized annulus fibrosus matrix hydrogels promote annulus fibrosus repair

**DOI:** 10.1016/j.bioactmat.2022.04.025

**Published:** 2022-05-10

**Authors:** Qiang Wei, Dachuan Liu, Genglei Chu, Qifan Yu, Zhao Liu, Jiaying Li, Qingchen Meng, Weishan Wang, Fengxuan Han, Bin Li

**Affiliations:** a Department of Orthopaedic Surgery, Orthopaedic Institute, The First Affiliated Hospital, Suzhou Medical College, Soochow University, Suzhou, Jiangsu, China; b Department of Orthopaedic Surgery, The First Affiliated Hospital, Shihezi University School of Medicine, Shihezi, Xinjiang, China; c Collaborative Innovation Center of Hematology, Soochow University, Suzhou, Jiangsu, China

**Keywords:** Decellularized matrix, Annulus fibrosus, Hydrogel, TGF-β1, Tissue repair

## Abstract

Annulus fibrosus (AF) repair remains a challenge because of its limited self-healing ability. Endogenous repair strategies combining scaffolds and growth factors show great promise in AF repair. Although the unique and beneficial characteristics of decellularized extracellular matrix (ECM) in tissue repair have been demonstrated, the poor mechanical property of ECM hydrogels largely hinders their applications in tissue regeneration. In the present study, we combined polyethylene glycol diacrylate (PEGDA) and decellularized annulus fibrosus matrix (DAFM) to develop an injectable, photocurable hydrogel for AF repair. We found that the addition of PEGDA markedly improved the mechanical strength of DAFM hydrogels while maintaining their porous structure. Transforming growth factor-β1 (TGF-β1) was further incorporated into PEGDA/DAFM hydrogels, and it could be continuously released from the hydrogel. The *in vitro* experiments showed that TGF-β1 facilitated the migration of AF cells. Furthermore, PEGDA/DAFM/TGF-β1 hydrogels supported the adhesion, proliferation, and increased ECM production of AF cells. *In vivo* repair performance of the hydrogels was assessed using a rat AF defect model. The results showed that the implantation of PEGDA/DAFM/TGF-β1 hydrogels effectively sealed the AF defect, prevented nucleus pulposus atrophy, retained disc height, and partially restored the biomechanical properties of disc. In addition, the implanted hydrogel was infiltrated by cells resembling AF cells and well integrated with adjacent AF tissue. In summary, findings from this study indicate that TGF-β1-supplemented DAFM hydrogels hold promise for AF repair.

## Highlights

• Injectable DAFM derived hydrogel with mechanical property matching natural AF and sustained TGF-β1 release was developed.
• DAFM derived hydrogels promote AF cell proliferation, migration and ECM production.
• DAFM derived hydrogels display good integration with host AF tissue.
• DAFM derived hydrogels facilitate AF repair and restore intervertebral disc biomechanics.

## Graphical abstract

**Figure:**
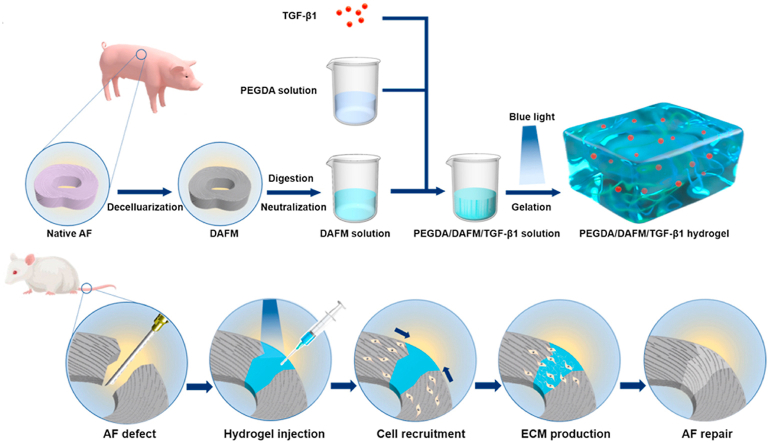

## 1 Introduction

Low back pain (LBP) is a prevalent musculoskeletal disorder which can lead to disability and impose a severe socio-economic burden [[1], [2], [3]]. Intervertebral disc (IVD) degeneration and disc herniation are the main causes of LBP [4,5]. Currently, the most common surgical procedure for disc herniation is discectomy, which alleviates clinical symptoms by removing the herniated nucleus pulposus (NP) tissue to relieve nerve compression. However, this procedure does not treat the annulus fibrosus (AF) defect, rendering the disc prone to reherniation, which occurs in 5–26% of postoperative patients [[6], [7], [8], [9]]. Furthermore, AF damage compromises the integrity of IVD as a load-bearing unit, resulting in mechanical imbalance, which may even exacerbate disc degeneration [[10], [11], [12]]. Therefore, it is necessary to repair AF in order to restore the disc function and reduce the incidence of recurrent herniation.

Many strategies have been proposed for the repair of AF defect. Although traditional AF repair devices can help restore mechanical stability, fixation of the device can cause damage to surrounding structures such as the cartilage endplate, and the long-term efficacy of the device is uncertain because of the absence of biological repair [13]. Recent studies show that tissue engineering presents favorable outcomes in the biological repair of AF. Studies have demonstrated that scaffolds loaded with AF cells or mesenchymal stem cells (MSCs) can facilitate AF repair [14,15]. However, such cell-based therapies may be limited by immunogenicity, undesired differentiation and tumorigenicity [16,17]. In addition to exogenous cell delivery, in situ tissue repair through cell recruitment and enhancement of cell function may be promising as a new therapeutic strategy.

Transforming growth factor-β1 (TGF-β1) is a potent growth factor (GF) that regulates cell proliferation, differentiation, and matrix production and acts a pivotal part in tissue repair and regeneration [18,19]. For example, TGF-β1 can achieve endogenous repair of cartilage by immunomodulatory regulation of macrophages, recruitment of MSCs, and promotion of chondrogenesis [20]. TGF-β1 can reduce autophagy and apoptosis of rat AF cells induced by oxidative stress through the ERK signaling pathway [21]. Moreover, a recent study has shown that the incorporation of TGF-β1 to implants induces the functional phenotype of AF cells and promotes cell proliferation and the synthesis of extracellular matrix (ECM) [22].

In addition to GFs, suitable biomaterials are essential for AF repair. The ideal repair material should possess good cytocompatibility, support cell migration, proliferation, ECM production, and be well integrated with surrounding tissues after implantation [23,24]. Many biomaterials have been developed for AF tissue engineering, such as collagen [15], fibrin [25], hyaluronic acid [26], alginate [27], and decellularized extracellular matrix (dECM) [14,28]. Among them, tissue-derived dECM stands out owing to its similarity in structure and composition to native tissue. The dECM is able to retain the GFs, matrix proteins, and glycosaminoglycans (GAGs) contained in native tissue and rebuild an environment similar to natural ECM for regeneration [[29], [30], [31]]. Moreover, dECM-derived hydrogels can facilitate the controlled-release and retention of GFs. Many GFs have a high affinity for the heparin sulfate proteoglycan of ECM, and this interaction enables ECM to act as a GF “storehouse” that can be released on demand to maintain tissue homeostasis or participate in tissue repair [32]. Despite these advantages, dECM-derived hydrogels have poor mechanical properties and degrade rapidly, which hinders their clinical application. AF exists in a complex mechanical environment; hence, the ability of repair materials to withstand the physiological pressure of the disc and maintain its structural integrity throughout treatment is critical to achieving AF regeneration [33]. In addition, injectable dECM-derived hydrogels which can be minimally implanted also have a unique advantage. The development of an injectable dECM-derived hydrogel with sufficient strength and fragmentation resistance through combination with synthetic polymers is a feasible route. Polyethylene glycol diacrylate (PEGDA) is a hydrophilic polymer with good biocompatibility that can be customized for the preparation of hydrogels [34]. When PEGDA is combined with dECM, the acrylate groups contained in PEGDA can cross-link with each other to entangle dECM components, resulting in dECM-derived hydrogels with higher mechanical strength.

**Scheme 1:**
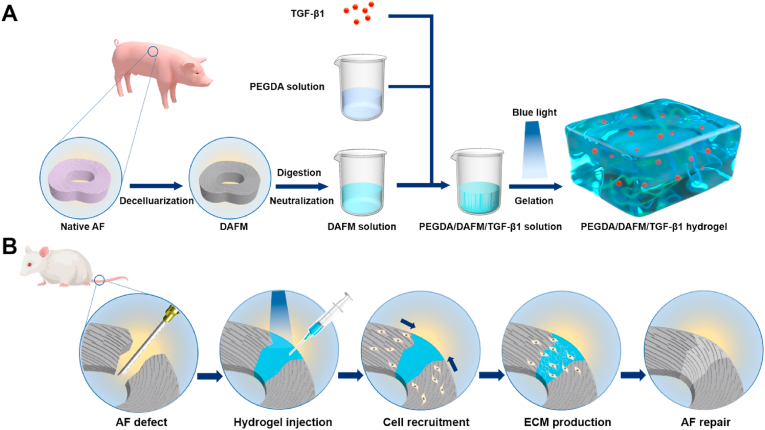
Summary of fabricating PEGDA/DAFM/TGF-β1 hydrogels for AF repair. (A) The detailed method and process of fabricating PEGDA/DAFM/TGF-β1 hydrogels. (B) The mechanism for AF repair by injecting hydrogels.

In the study, we aimed to develop a photocrosslinkable composite hydrogel based on PEGDA and decellularized annulus fibrosus matrix (DAFM), which has excellent mechanical stability. TGF-β1 was incorporated into the hydrogel to promote AF repair. The effects of the composite hydrogel, PEGDA/DAFM/TGF-β1, on the proliferation, migration, and ECM synthesis of AF cells were investigated *in vitro*. After the hydrogel was implanted into AF defect, its reparative effect was evaluated on the basis of gross, imaging, histological, and biomechanical findings (Scheme 1).

## 2 Materials and methods

### 2.1 Materials

Lithium phenyl (2, 4, 6-trimethylbenzoyl) phosphinate (LAP) was obtained from TCI Company, Japan. Triton X-100, pepsin and Collagen I were obtained from Sigma-Aldrich, USA. PEGDA was purchased from Alfa Aesar, USA. Collagenase was obtained from Yeasen Co., Ltd. (China). Ethanol, sodium hydroxide and acetic acid were obtained from Aladdin Co., Ltd. (China). The details of other reagents are given in the experimental methods.

### 2.2 Decelluarization of AF tissue

The DAFM was prepared following a previously reported protocol [35]. Briefly, AF tissues were collected from the fresh porcine spine. The samples were rinsed in phosphate buffered saline (PBS) and then cut into small pieces. The trimmed pieces were placed in 0.25% trypsin-PBS solution (Yuanye, China) and agitated at 37 °C for 24 h. Next, the tissue was washed by PBS and digested in nuclease solution (Yuanye, China) at 37 °C overnight. The tissue was placed in 1% Triton X-100 solution under agitation at least 24 h. Then, samples were rinsed in PBS for 6 cycles of 8 h each. Finally, the resulting DAFM was lyophilized and pulverized into powder and stored at −80 °C for future use.

### 2.3 Characterizations of DAFM

To evaluate the decellularization efficiency, the histological sections of native AF and DAFM were stained with hematoxylin and eosin (H&E, Yuanye, China) and DAPI (Invitrogen, USA) and the residual DNA was quantitatively measured with a Hoechst assay as previously described [36]. The main components in ECM such as collagen and GAGs were also quantitatively analyzed using GAGs assay kit (Hepengbio, China) and Hydroxyproline Content Assay Kit (Solarbio, China), respectively. The collagen content was determined based on the assumption that the hydroxyproline content was 13% of collagen [37].

### 2.4 Preparation of hydrogels

The DAFM powder was digested with pepsin acetic acid solution by stirring for 48 h to obtain 1% (w/v) DAFM solution. Undigested particles were removed by centrifugation. Then the digested solution was neutralized using NaOH solution at 4 °C. DAFM hydrogels were prepared by incubating the pre-gel solution at 37 °C for 30 min. For PEGDA/DAFM hydrogel preparation, the DAFM solution was homogeneously mixed with PEGDA and LAP. The final weight fractions of the LAP, DAFM and PEGDA in the solution were 0.25%, 1% and 15% (w/v), respectively. Then the PEGDA/DAFM pre-gel solution was crosslinked with blue light treatment (405 nm, 1 min). Similarly, the PEGDA/Collagen hydrogel was prepared as the control. PEGDA/DAFM hydrogels were prepared by adding TGF-β1 (PeproTech, USA) into the PEGDA/DAFM pre-gel solution at the concentration of 1 μg/mL.

### 2.5 Morphological characterizations of DAFM and PEGDA/DAFM hydrogels

DAFM and PEGDA/DAFM hydrogels were lyophilized with a vacuum freeze dryer. Then the microstructure of the lyophilized hydrogel was visualized by scanning electron microscopy (SEM) after spray coating with a thin layer of Au.

### 2.6 Mechanical tests

The mechanical properties of DAFM and PEGDA/DAFM hydrogels were determined by compressive tests using a universal testing instrument (HY-0580, Shanghai Hengyi Co., Ltd, China). All samples were prepared as standard cylinders of a height of 10 mm and a diameter of 4.5 mm. Four duplicates were set in each group. Stress-strain curves were generated by gradually applying compression load to the hydrogels (1 mm/min). The compressive modulus of all samples was calculated from the curves.

### 2.7 TGF-β1 release tests

To study the release profile of TGF-β1, the PEGDA/DAFM/TGF-β1 hydrogel was placed in PBS and kept shaking at 37 °C. The release of TGF-β1 at specific time points (0, 2 h, 6 h, 12 h, 1 d, 2 d, 3 d, 4 d, 5 d, 6 d, 7 d) was detected with an ELISA kit (Abclonal, USA).

### 2.8 Cell culture

All animal experiments followed the NIH Guide for the Care and Use of Laboratory Animals and were approved by the Institutional Animal Care and Use Committee of Soochow University. AF tissues were collected from rat tail IVDs. The tissue was trimmed and digested in collagenase solution (Yeasen, China). The digestion solution was filtered and centrifuged to collect cell pellets. The cells were cultured using DMEM/F12 complete medium after resuspension. All experiments were performed with AF cells at passage 2.

### 2.9 Chemotactic effect of TGF-β1

The chemotactic effect of TGF-β1 was evaluated using a Transwell chamber (8 μm pore size, Corning, USA). Briefly, the cells were starved overnight and treated with trypsin solution (Gibco, USA). Then the cells were seeded onto Transwell inserts at a density of 5 × 10^4^ per well of 24-well plate. The bottom chamber contained DMEM essential medium with different concentration of TGF-β1. After culture for 12 h, cells were fixed using 4% paraformaldehyde. Cells within the insert were removed, while those on the bottom surface were photographed and counted after crystal violet (Beyotime, China) staining.

### 2.10 Cell viability and proliferation tests

The PEGDA/Collagen hydrogels (PC group), PEGDA/DAFM hydrogels (PD group), and PEGDA/DAFM/TGF-β1 hydrogels (PDT group) with a diameter of 1.5 cm and a thickness of 0.5 cm were placed in a 24-well plate. AF cells were seeded on the surface of hydrogels at a density of 5 × 10^3^ per well. Three duplicates were set in each group. The viability of AF cells was estimated using a Calcein-AM/Ethidium homodimer-1 LIVE-DEAD cell staining kit (Invitrogen, USA). The cell images were captured using a fluorescence microscope. The proliferation of AF cells at 1, 4 and 7 days was determined by Cell Counting Kit-8 (CCK-8, Proteintech, USA) following the manufacturer's protocols.

### 2.11 Cell morphology observation

After crosslinking, three groups of hydrogels (PC, PD and PDT) with a diameter of 1.5 cm and a thickness of 0.5 cm were placed in a 24-well plate. AF cells were seeded on the surface of hydrogels at a density of 1 × 10^4^ per well. Three duplicates were set in each group. To evaluate the morphology of AF cells on hydrogels, the cells were fixed by paraformaldehyde. For SEM analysis, the constructs were dehydrated with gradient concentrations of ethanol and then treated with critical point drying. Finally, the samples treated with gold spraying were observed by SEM. For cytoskeleton staining, the samples were co-stained with a TRITC labeled probe (Yeasen, China) and DAPI. The samples were washed with PBS before being photographed with a fluorescence microscope (Carl Zeiss Inc, Thornwood, NY).

### 2.12 qRT-PCR analysis

Three groups of hydrogels (PC, PD and PDT) with a diameter of 3.4 cm and a thickness of 0.5 cm were placed in a 6-well plate. AF cells were seeded on the surface of hydrogels at a density of 2 × 10^5^ per well. Three duplicates were set in each group. After 14 days of culture on hydrogels, cells were collected. The extraction of total RNA was performed using a commercial kit (Vazyme, China) following instructions. The concentration and purity of RNA was determined using Nano Drop and then the RNA was reverse transcribed into cDNA using RT Master Mix (ABM, Canada). Finally, qRT-PCR was conducted to quantify the relative transcription levels of target genes (*Col1a1*, *Col2a1*, and *Acan*). The primers and genes are listed in Table S1.

### 2.13 Western blot analysis

Three groups of hydrogels (PC, PD and PDT) with a diameter of 3.4 cm and a thickness of 0.5 cm were placed in a 6-well plate. AF cells were seeded on the surface of hydrogels at a density of 2 × 10^5^ per well. Three duplicates were set in each group. Total protein of each sample was extracted using RIPA lysed buffer (Beyotime, China). Protein concentration of all samples was determined by a BCA Protein Assay Kit (Beyotime, China). The extracted proteins were separated by 10% SDS-PAGE gels before transfer to nitrocellulose membranes (Beyotime, China). Then the membranes were probed with primary antibodies (COL I, COL II, ACAN, and β-actin, Abcam, USA) after being blocked with skim milk. The next day, the membranes were incubated with the respective peroxidase-conjugated secondary antibody for 1 h. Finally, the proteins were visualized using a FluorChem imaging system.

### 2.14 Animal experiments

In the study, SD rats (12 weeks old, male) were divided into five groups: normal group, defect group, PEGDA/Collagen hydrogel injection group (PC group), PEGDA/DAFM hydrogel injection group (PD group) and PEGDA/DAFM/TGF-β1 hydrogel injection group (PDT group), respectively. Surgeries were carried out as previously described with minor modifications [15]. In brief, the rats were anesthetized with pentobarbital intraperitoneal injection and subsequently disinfected. The rat caudal vertebrae Co7/Co8 and Co8/Co9 were chosen as operation sites. After incision, one side of the AF was punctured with an 18 G needle to create a full-thickness defect without damaging NP. Hydrogels were implanted into AF defects with a syringe. After in situ curing using blue light, we sutured the surrounding tendons and muscles after implantation to help retain the hydrogel in the defect. Rats that underwent only puncture were defined as the defect group. The rats were sacrificed at 4 and 8 weeks after surgery and disc samples were collected.

### 2.15 Gross appearance assessment

Disc samples were collected and fixed with 10% formalin (Solarbio, China). After softening with decalcified fluid, samples were dissected with a blade along the line between the center of the disc and the initial site of puncture. The degree of disc degeneration was evaluated using Thompson score system based on the gross appearance [38].

### 2.16 Disc height measurement

The disc height of each sample was measured using Image J software from digital radiographs. The disc height was normalized to the average height of adjacent vertebrae to calculate the disc height index (DHI). The %DHI was defined as the ratio of the DHI in the operative groups to the DHI in the normal group.

### 2.17 MRI

The rat tail was examined by MRI at 4 and 8 weeks postoperatively. The water content of the operated disc was normalized to that of the normal disc to estimate the relative water content of NP according to the T2-weighted images. The modified Pfirrmann grading was used to evaluate disc degeneration.

### 2.18 Histology analysis

Rat tail samples obtained were fixed with 10% formalin. After decalcification and dehydration, the samples were embedded in paraffin. The paraffin sections were stained with H&E and safranin O-fast green (S&O) following instructions. Anti-COL I and Anti-COL II antibodies (Abcam, USA) were used for immunohistochemical staining according to our previous study [39].

### 2.19 Biomechanical tests

An axial compression test was used to assess the biomechanical properties of the IVD as previously described [11]. Briefly, the rat vertebra-IVD-vertebra motion segments were harvested after removing the surrounding soft tissues. The vertebrae of the samples were coated with bone cement to ensure their stability during the test. After preloading, the motion segment was placed on a dynamic mechanical analyzer for compression test. Four samples were tested in each group. A bilinear fitting compression curve was used to quantify toe and linear compressive modulus.

### 2.20 Statistical analysis

All date shown were mean ± standard deviation (SD). Statistical analyses were performed using ANOVA followed by Tukey's post hoc analysis (Graph prism software 7.0, USA). A statistically significant difference is considered if *p* is less than 0.05.

## 3 Results

### 3.1 Characterizations of DAFM

**Fig. 1:**
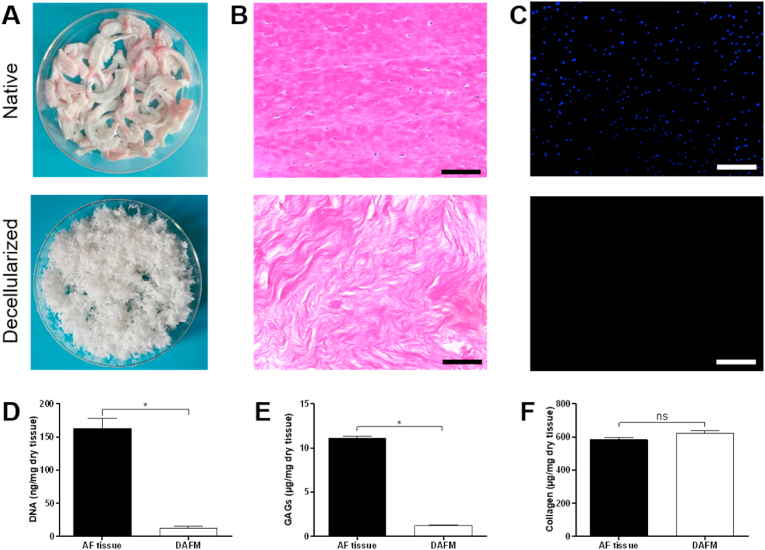
Characterizations of native and decellularized AF tissue. (A–C) Gross appearance, H&E and DAPI staining of AF tissue pre- and post decellularization. Scale bars, 100 μm. (D–F) DNA contents and ECM components of native and decellularized AF tissue. *, *p* < 0.05; ns, no significant difference.

Porcine AF tissue was decellularized by trypsin digestion followed by nuclease and Triton X-100 treatment. After decellularization, DAFM became a white and slightly loose substance (Fig. 1A). H&E staining results showed that most cells were removed, and DAPI staining confirmed the absence of nuclei (Fig. 1B and C). The content of DNA in DAFM was 13.1 ± 2.3 ng/mg dry tissue, which was significantly lower than that before decellularization (Fig. 1D). The key components of ECM, including collagen and GAGs, were also quantified. It was observed that GAG content in DAFM was significantly lost, accounting for only 11% of that before decellularization (Fig. 1E). Although there was no statistical difference in collagen content between DAFM and native tissue, the relative proportion of collagen in DAFM was slightly increased, which may be due to the change in components (Fig. 1F).

### 3.2 Characterizations of photocrosslinkable PEGDA/DAFM hydrogels

**Fig. 2:**
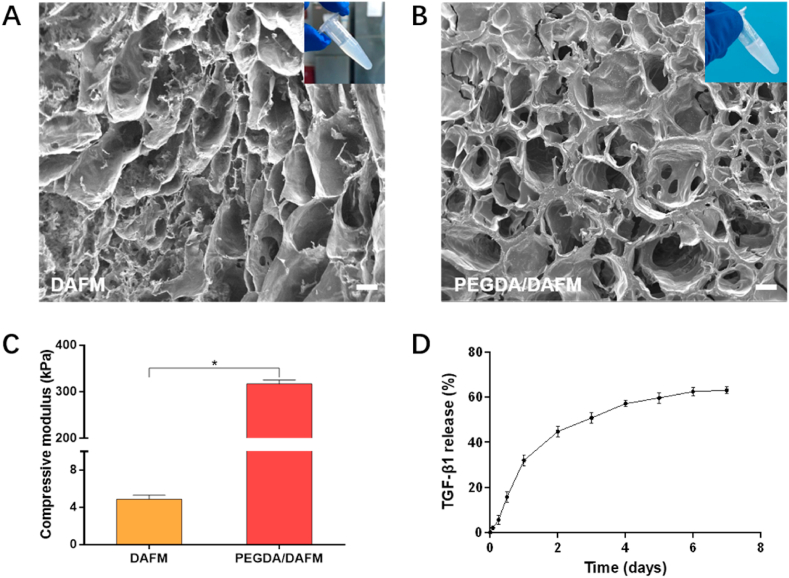
Characterizations of hydrogel microstructure and properties. (A, B) SEM observation of DAFM and PEGDA/DAFM hydrogels. Insets show the macroscopic images of hydrogels. Scale bars, 200 μm. (C) Compressive modulus of DAFM and PEGDA/DAFM hydrogels. (D) *In vitro* TGF-β1 release from the PEGDA/DAFM hydrogel. *, *p* < 0.05.

Although DAFM hydrogels well supported cellular activity, they could not provide stable structure due to lack of mechanical strength. To overcome this limitation, PEGDA was added into DAFM to prepare a photocrosslinkable hydrogel with good mechanical properties. After freeze drying, the morphological characteristics of DAFM and PEGDA/DAFM hydrogels were observed by SEM. The DAFM hydrogel presented a porous structure, with pore size roughly in the range of 10–600 μm (Fig. 2A). Compared with DAFM hydrogels, the pore size of PEGDA/DAFM hydrogels was smaller but more uniform (Fig. 2B). Moreover, it was observed that there were lots of interconnected micropores on the walls of PEGDA/DAFM hydrogels. Nutrients and metabolic products are more easily exchanged because of the presence of these internal channels. The mechanical performance of scaffolds acts a pivotal part in supporting cell adhesion and growth [40]. As shown in Fig. 2C, the compressive modulus of PEGDA/DAFM hydrogels (317.2 ± 8.2 kPa) was significantly higher than that of DAFM hydrogels (4.8 ± 0.4 kPa), and was close to the average compressive modulus of native AF [41], suggesting that the addition of PEGDA could enhance the mechanical properties of the hydrogels. The release behavior of TGF-β1 in PEGDA/DAFM hydrogel was evaluated by ELISA (Fig. 2D). Clearly, the release of TGF-β1 continuously happened and reached a plateau after 7 days.

### 3.3 Chemotactic effect of TGF-β1

Next, the chemotactic effect of TGF-β1 on AF cells was examined using a Transwell experiment. Compared with that in the control group (essential medium), there was a significant increase in the migration of AF cells through the Transwell membrane following the addition of TGF-β1 (Fig. S1A). The number of migrating cells increased with the amount of TGF-β1. Quantitative analysis showed that the chemotactic effect of TGF-β1 on AF cells was dose-dependent (Fig. S1B). The higher the concentration of TGF-β1, the more cells migrated.

### 3.4 Cell proliferation and morphology on hydrogels

**Fig. 3:**
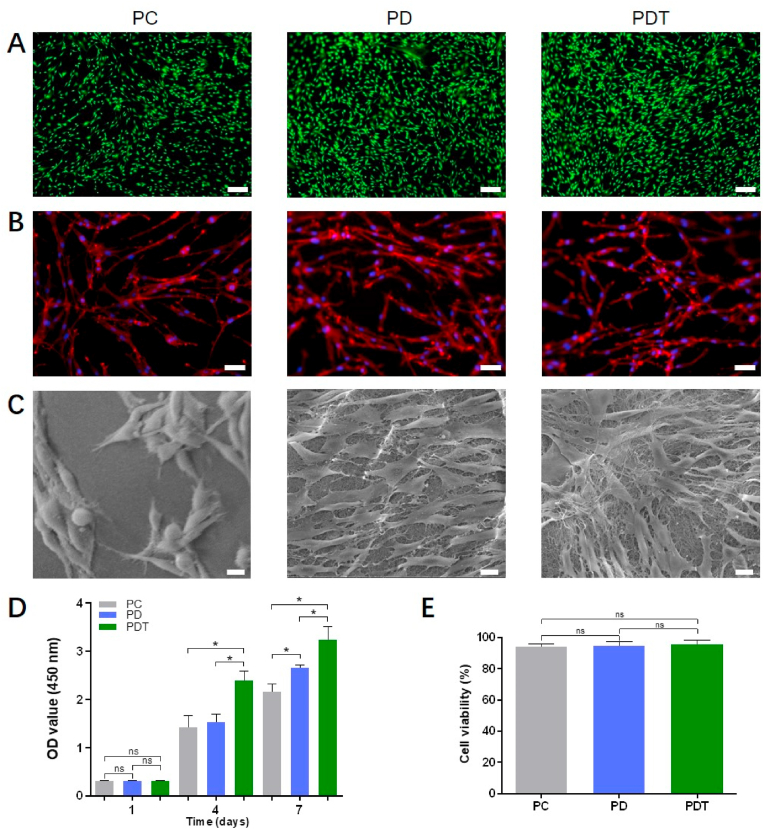
Growth and adhesion of AF cells on hydrogels. (A) Live/dead staining images of cells on PC, PD, and PDT hydrogels after 7 days. Scale bars, 200 μm. (B) F-actin staining images of AF cells at 2 days. Scale bars, 50 μm. (C) SEM images of AF cells at 2 days. Scale bars, 10 μm. (D) Cell proliferation. (E) Quantitative results of cell viability. *, *p* < 0.05; ns, no significant difference.

The cytotoxicity of PEGDA/Collagen, PEGDA/DAFM, and PEGDA/DAFM/TGF-β1 (marked as PC, PD, and PDT, respectively) hydrogels was assessed using live/dead staining (Fig. 3A). There were almost no dead cells on the hydrogels in all three groups, indicating that the hydrogels had no adverse effects on AF cells. The similar elongated spindle shape of AF cells in all three groups was observed from F-actin staining images (Fig. 3B). SEM images showed that the cell spreading in the PD and PDT groups was better than that in the PC group (Fig. 3C). Microfiber structures were observed on the hydrogel surface in the PD and PDT groups; these were not present in the PC group. The presence of these structures may be one of the factors that underlie the promoting effect of DAFM-derived hydrogels on cell adhesion and spread. The proliferation of AF cells on hydrogels was assessed using CCK-8 (Fig. 3D). On day 1, there was no substantial difference in cell proliferation between all groups. After 7 days, cell proliferation in the PD group was more extensive than in the PC group, indicating that DAFM was superior to collagen in terms of promoting the proliferation of AF cells. However, cell proliferation in the PD group was not as high as that in the PDT group after 4 and 7 days, indicating that the addition of TGF-β1 enhanced the proliferation of AF cells on DAFM. After 7 days of culture, no significant difference in cell viability was observed among the three groups (Fig. 3E).

### 3.5 ECM production

**Fig. 4:**
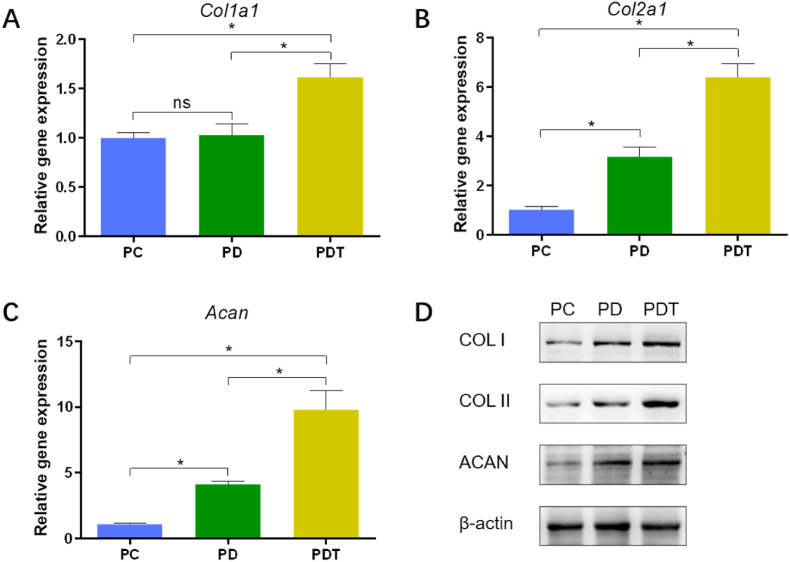
Effect of three groups of hydrogels on ECM production. (A) Gene expression of *Col1a1*, *Col2a1*, and *Acan* using *GAPDH* as housekeeping gene and the PC group as control. (B) Western blot analysis. *, *p* < 0.05; ns, no significant difference.

The expression of genes, including *Col1a1*, *Col2a1*, and *Acan,* was detected to evaluate the effect of three hydrogels on matrix production of AF cells (Fig. 4A–C). The expression level of *Col2a1* and *Acan* was higher in the PD group when compared to that in the PC group. Although there was no statistical difference, the expression level of *Col1a1* in the PD group (1.03 ± 0.12) showed a slightly increased trend compared with that in the PC group (1.00 ± 0.05). Among the three groups, the PDT group presented the highest expression level of matrix synthesis-related genes. Western blot analysis further confirmed that the production of COL I, COL II, and ACAN proteins was significantly enhanced in the PDT group compared with other two groups (Fig. 4D).

### 3.6 Efficacy of PEGDA/DAFM/TGF-β1 hydrogels for AF repair

#### 3.6.1 Gross morphology evaluation

**Fig. 5:**
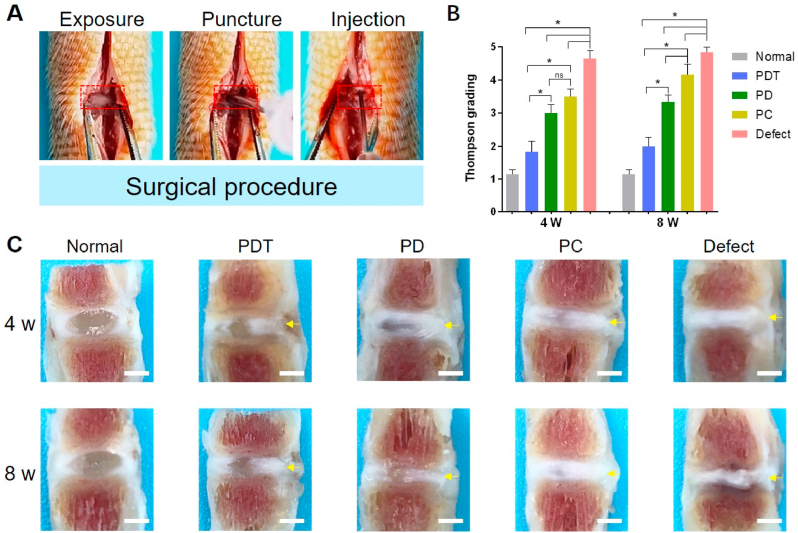
Macroscopic evaluation of *in vivo* repair effect. The red rectangles indicate the position of the disc. (A) Surgical procedures in a rat-tail needle puncture model. (B) Thompson grading system scores. (C) Gross images of AF defect repair. The yellow arrows denote the puncture sites. Scale bars, 1 mm *, *p* < 0.05; ns, no significant difference.

A rat-tail needle puncture model was established to assess the efficacy of PEGDA/DAFM/TGF-β1 hydrogels for AF repair (Fig. 5A). Under general anesthesia, the AF was exposed. The rats underwent puncture with an 18 G needle through a 1-cm longitudinal incision. Subsequently, PEGDA/Collagen, PEGDA/DAFM, or PEGDA/DAFM/TGF-β1 hydrogels were injected (at a volume of 10 μL) into the defect using a 26 G needle. After in situ photocrosslinking, the implanted hydrogel could be well maintained in the defect (Fig. S2). The gross morphologies of AF repair at 4 and 8 weeks after surgery are shown in Fig. 5C. Compared with the defect group, the discs in the hydrogel injection groups were significantly improved, which was reflected in the partial retention of the NP tissue to varying degrees and the prevention of disc collapse. In the PDT group, an obvious boundary between AF and NP was observed, which was not easy to discern in the PC and PD groups. After longitudinal comparison, it was found that the disc changes in the PDT and PD groups were not significant, while the disc in the PC group showed obvious NP atrophy. The Thompson score also demonstrated that the repair effect of PDT group was better than the other two groups at 4 and 8 weeks (Fig. 5B).

#### 3.6.2 Imaging evaluation

**Fig. 6:**
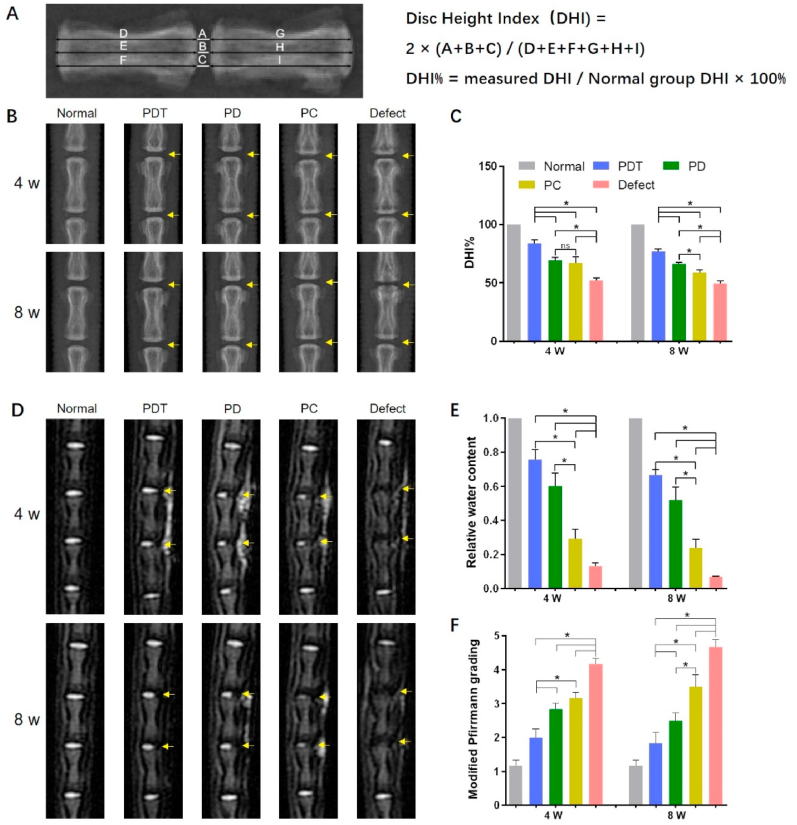
Imaging evaluation of AF repair. (A) Diagram demonstrating the calculation for Disc Height Index (DHI) and DHI%. (B) Typical radiographs of the rat tail in five groups. (C) Quantitative analysis of disc height. (D) Typical T2-weighted images of the rat tail in five groups. (E) Quantitative results of water content in the NP tissue. (F) Modified Pfirrmann grading. The yellow arrows denote the puncture sites. *, *p* < 0.05; ns, no significant difference.

Changes in the disc height were assessed based on obtained radiographs (Fig. 6A). In the defect group, the disc collapsed significantly (Fig. 6B). At 8 weeks, damage to the adjacent vertebrae was observed in the defect group. Among the hydrogel injection groups, the PDT group performed best in terms of maintaining disc height (Fig. 6C). The disc height in the PD group was slightly higher than that in the PC group at 8 weeks. As shown in Fig. 6D, the defect group presented significant loss of hydration compared with hydrogel injection groups. The defect group showed black discs as early as 4 weeks, indicating severe disc degeneration. At 4 weeks, the NP signal intensity of operated discs in the PDT group was close to that of normal NP. Overall, the relative water content of the NP after surgery showed a decreasing trend over time (Fig. 6E). The higher relative water content was observed in the PD group compared with the PC group at 4 and 8 weeks, and the modified Pfirrmann grading of the PD group was better when compared to the PC group at 8 weeks, indicating that DAFM was superior to collagen in terms of promoting AF repair and alleviating disc degeneration (Fig. 6F). Interestingly, the injected hydrogels showed T2-weighted signals similar to that of NP on T2-weighted images. Although the signals could be clearly observed at 4 weeks, most of them disappeared at 8 weeks, indicating that the materials began to degrade over time.

#### 3.6.3 Histology evaluation

**Fig. 7:**
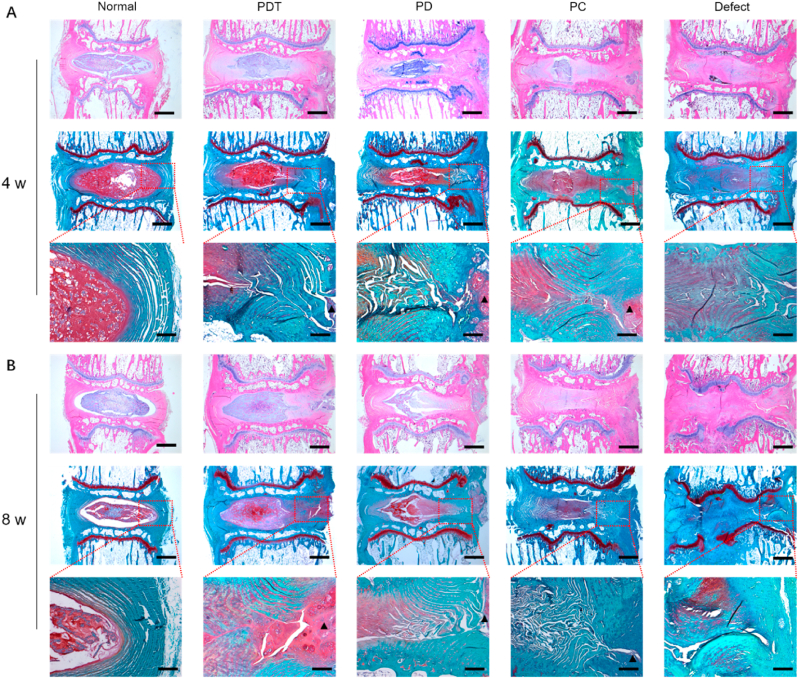
Histology evaluation of AF repair by H&E and S&O staining at (A) 4 and (B) 8 weeks after surgery. The red rectangles in the second line denote the zones where the higher magnification images in the third line were captured. The black triangles represent the areas where the hydrogels were implanted. Scale bars, 1 mm (1^st^ and 2^nd^ rows in each set) or 250 μm (3^rd^ row).

The repair effect on the AF defect was assessed using H&E and S&O staining (Fig. 7A and B). The defect group showed obvious defect space and almost no NP tissue at 4 and 8 weeks. The absence of NP led to the conduction of mechanical load directly on the endplate, which may have contributed to the endplate damage in the defect group at 8 weeks. At 8 weeks, although the annulus defect space was significantly narrowed in the PDT group, obvious gaps were still observed in the remaining groups together with disordered surrounding annulus tissue. In addition, the hydrogel in the PDT group was infiltrated by cells, most of which appeared at the conjugation of the hydrogel and adjacent tissue, and the cell morphology was similar to that of AF cells. These results demonstrate the progressive integration of PEGDA/DAFM/TGF-β1 hydrogels with host tissue after implantation. Immunohistochemical staining showed that PDT group exhibited the largest positive area for COL I and COL II compared with the other three groups, suggesting that the implantation of PEGDA/DAFM/TGF-β1 hydrogels delayed the degeneration of IVD (Fig. S3).

#### 3.6.4 Biomechanical evaluation

**Fig. 8:**
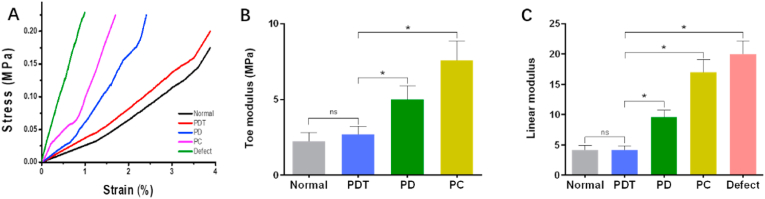
Biomechanical evaluation of AF repair at 8 weeks after surgery. (A) Typical stress-strain curves of rat tail motion segments for each group. (B) Toe modulus. (C) Linear modulus. *, *p* < 0.05; ns, no significant difference.

The rat tail IVD is a common model for biomechanical evaluation because its biomechanical parameters are similar to that of human lumbar IVDs when normalized by geometry [11,42]. To assess the changes in the biomechanics of the disc after surgery, axial compression tests were performed for the whole motion segments consisting of vertebra-IVD-vertebra. The mechanics of the toe and the linear region are mainly dominated by the function of the NP and AF, respectively [43]. As shown in Fig. 8A, representative stress-strain curves for each group demonstrate changes in IVD mechanical function. In the defect group, toe modulus could not be measured because of the absence of NP (Fig. 8B). Toe modulus of PDT group was close to that of normal group, and markedly lower than that of the PD and PC groups. In terms of linear modulus, no significant difference was observed between the PDT group and the normal group (Fig. 8C).

## 4 Discussion

In this study, an injectable photocrosslinkable PEGDA/DAFM hydrogel that sustainably released TGF-β1 was prepared. H&E and DAPI staining results indicate that the decellularization process is successful, and this is further confirmed by subsequent DNA assay. Although the amount of GAGs decreased after decellularization, most of the collagen was retained. The loss of GAGs may be related to its high solubility and small molecular size [44].

Good mechanical properties of the biomaterials are critical for effective AF repair. Weak strength could be one of the reasons for implantation failure, because the biomaterials are not able to withstand the intradiscal pressure and radial stress of the annulus tissue [45]. ECM-based hydrogels have unique advantages in tissue repair. However, they are inherently fragile and prone to structural changes when seeded with cells, which limited their application in tissue repair. The addition of PEGDA significantly improved the mechanical properties of DAFM hydrogels while retaining the porous structure with pore sizes of about 200 μm. It has been reported that pore sizes in the range of 150–500 μm are favorable for tissue ingrowth and differentiation of fibrocartilage [46,47]. In addition, the PEGDA/DAFM hydrogels developed in this study were injectable and photocurable, and could be implanted into defects in a minimally invasive manner.

Delivery of exogenous cells to AF defects has been shown to promote AF regeneration [15,48,49]. However, the safety problems associated with cell transplantation have not been solved [[50], [51], [52], [53]]. Endogenous repair strategies are beginning to attract the attention of researchers. An alginate-collagen composite scaffold with shape-memory behavior has been developed to enhance the migration of AF cells and have shown potential to repair AF *in vitro* and *ex vivo*, but have not been verified *in vivo* [54]. A recent study used a fibrin gel containing CCL5 to recruit AF cells for annulus repair [55]. *In vitro* investigation demonstrated the ability of CCL5 to promote AF cell migration, however, no significant AF repair effect was observed *in vivo.* This suggests that, in addition to chemokines, appropriate scaffolds should also be considered to provide a favorable microenvironment for the recruited cells when designing an endogenous repair strategy. The dECM may be the best choice in terms of providing a microenvironment similar to natural tissue for the recruited cells, considering that no natural or artificial scaffolds can mimic all the characteristics of native ECM [36]. In addition, ECM-based hydrogels have been shown to function as GF reservoirs [32]. In this study, TGF-β1 could be continuously released from PEGDA/DAFM hydrogels and exhibited a chemotactic function on AF cells. The cells seeded on hydrogels in the three groups showed good biocompatibility. Cells in the PDT group proliferated more rapidly and showed higher expression levels of matrix synthesis-related genes and proteins. These results suggest that TGF-β1-supplemented DAFM hydrogels not only induce cell migration, but also promote the deposition of ECM by increasing the potential of recruited cells. It's worth noting that no significant difference existed in the gene expression of *Col1a1* between PC and PD groups. However, it is clear, from Western blot analysis, that the protein level of COL1 in PD group was higher than that in PC group, indicating that production of COL1 was increased when cells were cultured in a tissue derived ECM microenvironment (Fig. 4D). In fact, the process of transcription and translation in cells is influenced by a variety of factors. The production of a certain protein is not determined by RNA alone; other factors, including the half-life and synthetic rate of the protein, also affect the level of protein production. Therefore, it is not uncommon that there is inconsistency between gene expression and protein level.

The overall results of the study show that the AF repair effect in the PDT group is significantly better than that in the other three groups and close to that in the normal group. Gross findings and scores showed minimal atrophy of NP and a clear boundary between NP and AF in the PDT group. Compared with the PC group, the PD group could restore disc height and slow down disc degeneration better, suggesting that DAFM was superior to collagen in terms of AF repair, which was similar to the results of Peng et al. [14]. Derived from native tissues, dECM well preserves the native ECM components, including collagen, glycosaminoglycan, and GFs [30]. Therefore, the components of dECM are more similar to native tissues compared to that of collagen alone. Another important feature of dECM that distinguishes it from collagen is the diversity of its structure and spatial distribution of functional components [56]. As a result, dECM enables better recapitulation of tissue-specific microenvironmental niches [57]. A large number of studies have demonstrated that dECM is superior to collagen in the regeneration of tissues such as bone [58], spinal cord [59], and meniscus [37]. According to the modified Pfirrmann grading, DAFM hydrogels loaded with TGF-β1 significantly alleviated disc degeneration compared with the other three groups.

Because of NP swelling pressure, the ability of the implanted scaffold to repair the inner AF defect is limited [60]. As a result, full-thickness AF repair has been a challenge. In recent years, some biomaterials have been applied in the field of AF repair. In previous research, high-density collagen crosslinked with riboflavin has been implanted into AF defects to form a fibrous cap in the outer AF zone, which prevented NP atrophy and maintained disc height [15,61]. DAFM hydrogels crosslinked with genipin were developed to promote AF repair by directing the differentiation of encapsulated MSCs towards AF cells [14]. However, the defect in the inner AF remained unrepaired in these studies, providing NP tissue with a path of least resistance if it herniates again [16]. Keeping the implanted hydrogel to stably stay in the defect is the key for it to integrate with host tissue and repair the AF defect. It is noteworthy that in the PDT group, the AF defect gap, including the AF inner region, almost disappeared at 8 weeks. PEGDA/DAFM/TGF-β1 hydrogels not only retained at the defect, but also achieved good integration with surrounding host tissues. Many cells similar in morphology to the AF cells appeared at the site where the hydrogel was attached to the surrounding tissue. This finding further demonstrates the ability of TGF-β1-supplemented DAFM hydrogels to promote the migration of AF cells.

Selection of appropriate animal models is very important for evaluating the overall repair effect and functional restoration of intervertebral discs (IVDs). However, none of the existing animal models is ideal because the IVDs of animals are not loaded exactly the same way as human IVDs do [45]. The rat caudal disc model has been widely used in AF tissue engineering in light of the ease and reproducibility of surgeries, relatively low cost and moderate mechanical loading level on caudal discs [[62], [63], [64]]. More importantly, there are similarity in the mechanical performance of disc compression and torsion between rodents and humans when normalized for geometry, implying that rat IVDs can serve as a useful model to mechanically mimicking human IVDs [65]. The function of natural IVD is mainly reflected in mechanics, supporting the compression load on the spine by hydrostatic pressure in the NP and straining the AF fibers under circumferential stress [43]. The ideal strategy for AF repair should restore disc mechanics to maintain normal spine function and slow the progression of disc degeneration [6]. Reduced water content suggests NP fibrosis, a sign of IVD degeneration [66]. In this study, the toe modulus in the PDT group was closest to that in the normal group. This may be due to the minimal reduction of water content in the PDT group and no apparent collapse of the inner AF into the NP region. Furthermore, the linear modulus in the PDT group was similar to that in the normal group, suggesting that PEGDA/DAFM/TGF-β1 hydrogels functionally integrated with the surrounding AF tissue and helped restore the IVD biomechanics.

Despite the exciting results, there are limitations in this study. Because of the lack of specific markers for AF cells [67], the infiltrated cells in the scaffold could not be identified as AF cells, although they were morphologically similar to AF cells. We are currently exploring the use of single-cell sequencing to analyze various functional subgroups of AF cells and screen for appropriate specific markers. Further studies are also needed to explore the underlying mechanisms by which DAFM regulates the behaviors of AF cells and promotes AF regeneration.

## 5 Conclusion

In this study, a photocrosslinkable and TGF-β1-supplemented DAFM-derived hydrogel has been successfully developed for AF repair. This hydrogel provided desired injectability and structural and mechanical stability, and the tissue-specific microenvironment that could promote cellular activities and tissue regeneration of AF. Further, the sustained release of TGF-β1 from the hydrogel promoted the migration of AF cells, which facilitated the integration of hydrogels with host AF tissue, prevented NP atrophy, retained disc height, and restored the disc biomechanics. Together, findings from this study indicate that the TGF-β1-supplemented DAFM hydrogel might be an ideal candidate of scaffolds for AF tissue engineering applications.

## CRediT authorship contribution statement

**Qiang Wei:** Conceptualization, Methodology, Validation, Formal analysis, Investigation, Data curation, Writing – original draft, Writing – review & editing, Visualization. **Dachuan Liu:** Validation, Formal analysis, Investigation, Writing – review & editing. **Genglei Chu:** Conceptualization, Methodology, Writing – review & editing. **Qifan Yu:** Validation, Formal analysis, Investigation. **Zhao Liu:** Visualization. **Jiaying Li:** Methodology, Investigation. **Qingchen Meng:** Formal analysis, Investigation. **Weishan Wang:** Methodology, Formal analysis, Writing – review & editing. **Fengxuan Han:** Conceptualization, Formal analysis, Funding acquisition, Writing – review & editing. **Bin Li:** Conceptualization, Writing – review & editing, Visualization, Supervision, Project administration, Funding acquisition.

## Declaration of interests

The authors declare that they have no known competing financial interests or personal relationships that could have appeared to influence the work reported in this paper.

## Abbreviations

LBP: low back pain
IVD: intervertebral disc
NP: nucleus pulposus
AF: annulus fibrosus
DAFM: decellularized annulus fibrosus matrix
MSCs: mesenchymal stem cells
GF: growth factor
ECM: extracellular matrix
dECM: decellularized extracellular matrix
GAG: glycosaminoglycan
PEGDA: polyethylene glycol diacrylate
LAP: lithium phenyl (2, 4, 6-trimethylbenzoyl) phosphinate
PBS: phosphate buffered saline
TRITC: tetramethylrhodamine
SEM: scanning electron microscopy
H&E: hematoxylin and eosin
S&O: safranin O-fast green
DHI: disc height index
