# Supplementary material for: TGF-β1-supplemented decellularized annulus fibrosus matrix hydrogels promote annulus fibrosus repair
Source: Bioact Mater. 2022 May 10;19:581–93. doi: 10.1016/j.bioactmat.2022.04.025 (PMC9108517; doi:10.1016/j.bioactmat.2022.04.025)
Supplement: Multimedia component 1 [file mmc1.docx]

**Supplementary materials**

**Table S1**. Primer sequences used for qRT-PCR.

| **Primer** | **Sequence (forward 5'-3')** | **Sequence (reverse 5'-3')** |
| --- | --- | --- |
| ***Col1a1***  ***Col21***  ***Acan***  ***GAPDH*** | TGTTGGTCCTGCTGGCAAGAATG  ACGCTCAAGTCGCTGAACAACC  GCTACGACGCCATCTGCTACAC  GACATGCCGCCTGGAGAAAC | GTCACCTTGTTCGCCTGTCTCAC  ATCCAGTAGTCTCCGCTCTTCCAC  AGTCCTCTTCACCACCCACTCC  AGCCCAGGATGCCCTTTAGT |


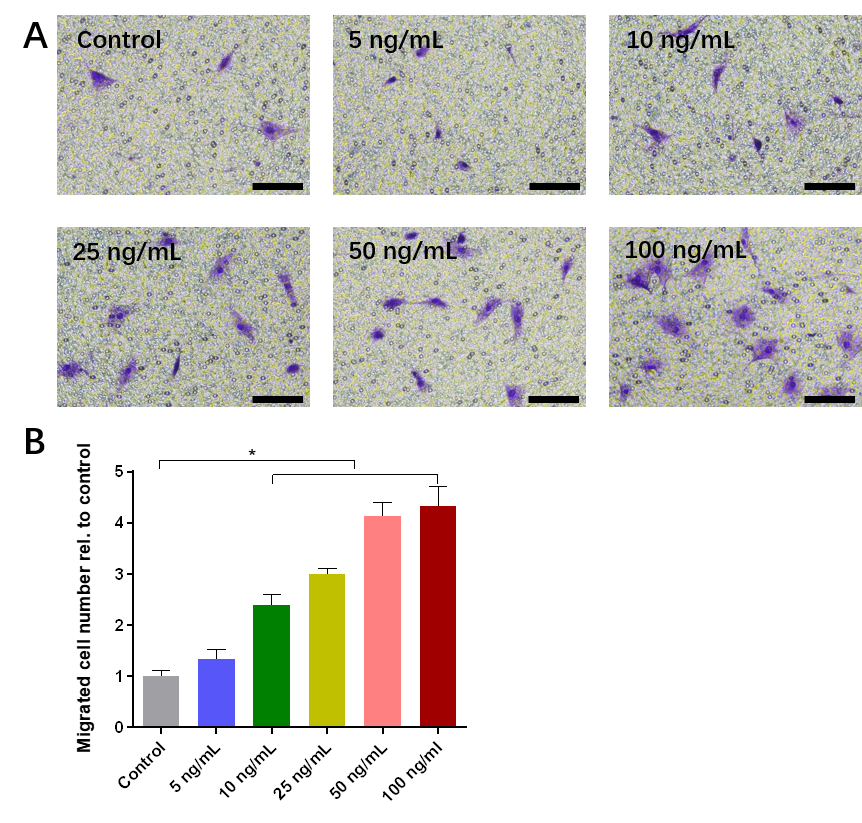


Fig. S1. Chemotactic effect of TGF-β1. (A) Migration of AF cells treated with different concentrations of TGF-β1 in Transwell migration assay. The migrating cells are spotted using crystal violet staining. Scale bars, 100 μm. (B) Quantitative comparison of the number of AF cells migrated through the Transwell membrane. Migrating cell numbers are normalized to that of control group. *, *p* < 0.05.


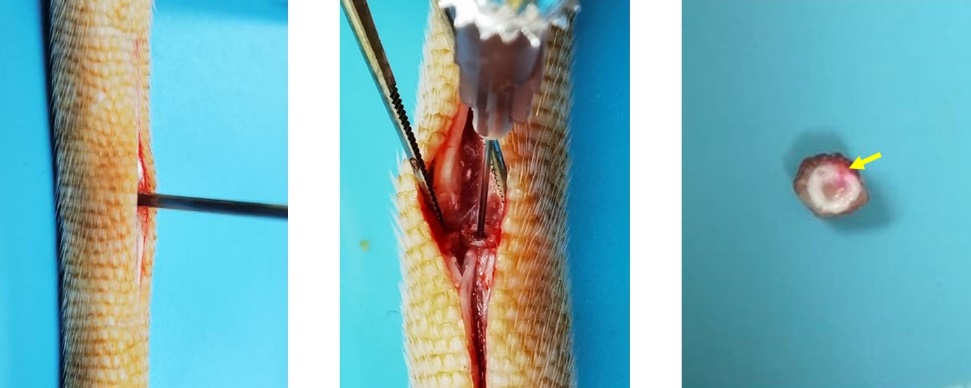


Fig. S2. Schematic of injecting the hydrogel into the AF defect after puncture. The PEGDA/DAFM hydrogel was dyed red to track its presence in the disc. The yellow arrow denotes the hydrogel position.


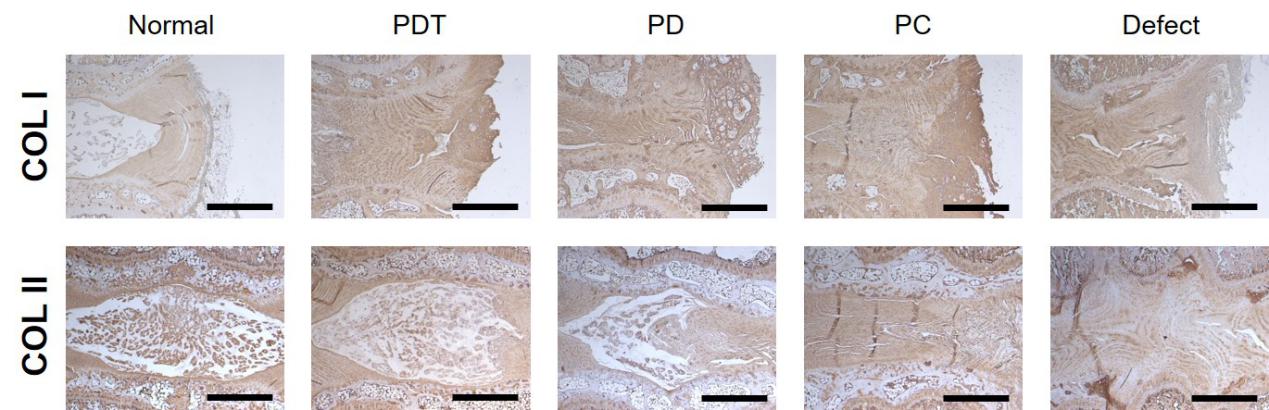


Fig. S3. Immunohistochemical staining of COL I and COL II at 8 weeks. Scale bars, 1 mm.
